# Evaluation of Salivary Pepsin Levels and Dental Erosion in Patients With Gastroesophageal Reflux Disease

**DOI:** 10.7759/cureus.34744

**Published:** 2023-02-07

**Authors:** Yousif S Rajab, Taghreed F Zaidan

**Affiliations:** 1 Department of Oral Diagnosis, College of Dentistry, University of Baghdad, Baghdad, IRQ; 2 Department of Dentistry, Al-Turath University College, Baghdad, IRQ

**Keywords:** gerd, salivary ph, gastroesophageal reflux disease, dental erosion, salivary pepsin

## Abstract

Background

Gastroesophageal reflux disease (GERD) is a common gastrointestinal condition affecting many individuals worldwide. GERD is characterized by esophageal symptoms and may contribute to various extraesophageal symptoms, including dental erosion (DE). This study aimed to estimate the levels of pepsin in the saliva of GERD patients to evaluate and compare the activity of pepsin between the GERD and healthy groups and investigate the prevalence of DE in the GERD group.

Methodology

In this case-control study, 40 patients with GERD, diagnosed with an endoscope, and 35 healthy subjects were included. Patients and healthy individuals were subjected to the GERD questionnaire (GerdQ). A dental assessment was performed using the Basic Erosive Wear Examination (BEWE). A total score of the BEWE risk level was obtained by summing the scores obtained in each sextant (no risk = ≤2, low risk = 3-8, medium risk = 9-13, and high risk = >13). Five milliliters of unstimulated saliva were collected in a sterile tube. The salivary pepsin levels examined using the enzyme-linked immunosorbent assay were recorded for both groups.

Results

DE was significantly more frequent in GERD patients compared to healthy subjects. Overall, 32 (80%) GERD patients and 11 (31.4%) healthy individuals had DE risk. The mean salivary pepsin was 43.60 ± 10.61 ng/mL in the GERD group and 20.60 ± 9.27 ng/mL in the healthy group. A statistically significant difference was found in pepsin levels between the two groups (p < 0.001).

Conclusions

The study concluded that GERD patients had a higher prevalence of DE than healthy individuals. Our findings suggest that elevated salivary pepsin levels and their role as a factor responsible for DE need further evaluation to understand the mechanisms of pepsin-mediated damage leading to DE.

## Introduction

Gastroesophageal reflux disease (GERD) is a common gastrointestinal disorder caused by uncontrolled and repeated backward reflux of gastric contents into the esophagus [1]. This leads to typical symptoms of troublesome heartburn and regurgitation. The disease can also lead to associated extraesophageal symptoms of cough, laryngitis, asthma, and dental erosion (DE). In addition, it may result in complications of esophagitis, strictures, and Barrett’s esophagus [2]. The factors contributing to GERD may include increased frequency of transient lower esophageal sphincter relaxation (TLESR), low pressure of lower esophageal sphincter, delayed gastric emptying, hiatal hernia, the presence of an acid pocket, and ineffective esophageal clearance [3].

Esophageal mucosal damage is caused by exposure of the esophagus to an excessive amount of acid, which can be inhibited by proton pump inhibitors (PPIs) [4]. When extraesophageal symptoms develop, the results of using PPI medications are poor; hence, extraesophageal reflux symptoms are much more likely to be mediated by pepsin than acids [5].

Pepsin is a proteolytic enzyme secreted from chief cells within the stomach as pepsinogen, which is then activated by hydrochloric acid. When pepsin is detected in regions other than the stomach (such as the throat or mouth), it indicates that it has been refluxed into that region [6]. Salivary pepsin levels have been shown to be associated with esophageal mucosal damage [7], but a few studies have reported salivary pepsin and oral findings in GERD patients.

DE is a non-carious lesion, and demineralization of dental hard tissues induced chemically by non-bacteriogenic acids may lead to dentin exposure and hypersensitivity [8]. These acids may be from extrinsic sources (such as diet) or intrinsic sources (such as reflux and vomiting) [9].

The aim of this study was to evaluate pepsin levels in the saliva of GERD patients and assess the prevalence of DE in patients with GERD.

## Materials and methods

The case-control study was conducted at the Department of Gastroenterology, Al Ramadi Teaching Hospital, Iraq, from December 2021 until June 2022. It was approved by the Research Ethics Committee of the College of Dentistry, University of Baghdad with reference number 439 (project number 439721 dated 4.8.2022). All subjects provided written informed consent after obtaining a complete and clear explanation of the study’s aims prior to recruitment. A total of 75 participants were included in this study and divided into two groups. Group I (the GERD group) consisted of 40 patients whose clinical and endoscopic examinations confirmed the presence of GERD. Group II (the control group) comprised 35 subjects as healthy controls who had no clinical evidence of GERD symptoms or other gastrointestinal tract disorders.

The inclusion criteria of this study were male and female patients of any age with a clinically and endoscopically established diagnosis of GERD. We excluded patients on antacids or PPI therapy for a period of two weeks before taking samples, pregnant women, and patients with a history of esophageal or gastric surgery. A verified questionnaire, requiring data from the individuals about their age, gender, occupation, pregnancy for females, medical history, and family history, was given to all subjects included in this study. Each participant completed a GERD questionnaire (GerdQ) [10]. The GerdQ is a diagnostic tool that helps diagnose GERD symptoms. The six questions of GerdQ assess four positive predictors of GERD (regurgitation, heartburn, the use of over-the-counter medications, and sleep disturbance) and two negative predictors of GERD (nausea and epigastric pain). Each predictor is rated with an ascending score for positive predictors (from 0 to 3) but a descending score for negative predictors (from 3 to 0) on a Likert scale (0 = 0 day, 1 = 1 day, 2 = 2-3 days, and 3 = 4-7 days). The overall total score (0-18) was calculated, and a GERD diagnosis was made using a cut-off score of >8 [10]. All patients had already undergone the endoscopic examination.

DE was clinically evaluated using the Basic Erosive Wear Examination (BEWE) index [11]. The BEWE index comprises a four-level scale (0 = no surface loss; 1 = initial surface loss; 2 = distinct defect, surface loss < 50%; 3 = hard tissue loss, surface loss > 50%). In each sextant, the tooth surface that showed the highest value was recorded as a sextant score. A total score of the risk level for each patient was obtained by summing the scores obtained in each sextant (no risk = ≤2, low risk = 3-8, medium risk = 9-13, and high risk = >13) [11].

Fasting, unstimulated saliva was collected in sterile tubes, and the pH of the saliva was evaluated immediately using a pH meter. Saliva samples were then placed in sterile plastic tubes containing 0.5 mL of 0.01 mol/L citric acid (pH 2.5) and refrigerated at 4°C until analysis. Pepsin was measured using a Human Pepsin (PP) enzyme-linked immunosorbent assay (ELISA) kit from Shanghai YL Biotech Co., Ltd.

Statistical analysis

The data collected were entered into a Microsoft Excel sheet and analyzed using SPSS software version 28 (IBM Corp., Armonk, NY, USA). Descriptive statistics included mean and standard deviation, and Student’s t-test was used for comparing mean values. Pearson’s chi-square test (χ^2^ test) tested the significant difference in percentages. Statistical significance was considered whenever the p-value was equal to or less than 0.05.

## Results

The mean age of the GERD patients was 34.6 years, with a standard deviation of 8.6 years (n = 40). The mean age of healthy subjects was 32.9, with a standard deviation of 8.1 (n = 35). Statistical analysis showed no significant difference between groups regarding age (p = 0.38). Overall, 45% (n = 18) of the GERD patients were male, and 55% (n = 22) were female. There were no significant differences between the study groups according to gender distribution (p = 0.42) (Table 1).

**Table 1:** Comparison of age and gender between the study groups. GERD = gastroesophageal reflux disease; SD = standard deviation; N = number; % = percentage

Parameters	GERD	Control	P-value
Age (mean ± SD)	34.6 ± 8.6	32.9 ± 8.1	0.38
Male (N, %)	18 (45%)	19 (54.3%)	0.42
Female (N, %)	22 (55%)	16 (45.7%)	

Regarding dietary habits, statistical analysis showed no significant difference between studied groups for tea, coffee, and carbonated beverage intake, while the result showed a significant difference between studied groups regarding vinegar and spicy food consumption (Table 2).

**Table 2:** Comparison of dietary habits among patients with GERD and controls. GERD = gastroesophageal reflux disease; N = number; % = percentage; * = statistical significance (p ≤ 0.05)

Dietary habits ≥1 per day		GERD (n = 40)	Control (n = 35)	P-value
		N (%)	N (%)	
Tea	Yes	30 (75)	29 (82.9)	0.40
	No	10 (25)	6 (17.1)	
Coffee	Yes	6 (15)	3 (8.6)	0.39
	No	34 (85)	32 (91.4)	
Vinegar	Yes	18 (45)	6 (17.1)	0.01*
	No	22 (55)	29 (82.9)	
Spicy food	Yes	9 (22.5)	2 (5.7%)	0.04*
	No	31 (77.5)	33 (94.3)	
carbonated beverage	Yes	22 (55)	23 (65.7)	0.34
	No	18 (45)	12 (43.3)	

Our study showed that eight (20%) GERD patients had no risk of DE, and 32 (80%) patients had a risk of DE (52.5% had low risk, and 27.5% had medium risk). No subjects in either group were at a high risk of DE. Statistical analysis showed a significant difference in DE between the GERD and control groups (p < 0.001) (Table 3).

**Table 3:** Prevalence of DE according to BEWE score in GERD patients and control group. DE = dental erosion; GERD = gastroesophageal reflux disease; BEWE = Basic Erosive Wear Examination; N = number; % = percentage; * = statistical significance (p ≤ 0.05)

BEWE risk levels	GERD (N = 40)	Control (N = 35)	P-value
	N (%)	N (%)	
None (≤2)	8 (20%)	24 (68.6%)	<0.001*
Low (3–8)	21 (52.5%)	9 (25.7%)	
Medium (9–13)	11 (27.5%)	2 (5.7%)	

The mean salivary pH value in the GERD group was 6.95, with a standard deviation of 0.25. In control subjects, the mean salivary pH was 7.11, with a standard deviation of 0.17. Statistical analysis using the t-test showed a statistical difference between the study groups (p = 0.002). Salivary pH was significantly decreased in GERD patients (Table 4).

**Table 4:** Comparison of mean salivary pepsin level and mean salivary pH between the study groups. GERD = gastroesophageal reflux disease; SD = standard deviation; * = statistical significance (p ≤ 0.05)

Parameters	GERD	Control	P-value
Salivary pH (Mean ± SD)	6.95 ± 0.25	7.11 ± 0.17	0.002*
Salivary pepsin (ng/mL) (Mean ± SD)	43.60 ± 10.61	20.60 ± 9.27	<0.001*

**Figure 1:**
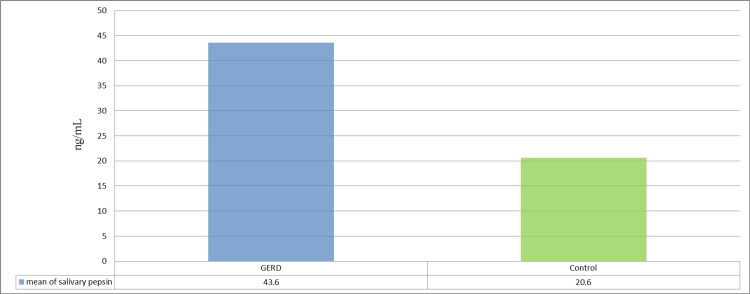
Comparison of mean salivary pepsin level between the GERD and control groups. GERD = gastroesophageal reflux disease

The mean salivary pepsin in the GERD group was 43.60 ng/mL, with a standard deviation of 10.61 ng/mL. The mean salivary pepsin in the control group was 20.60 ng/mL, with a standard deviation of 9.27 ng/mL. Statistical analysis using the t-test showed a statistical difference between study groups (p < 0.001) (Table 4, Figure 1).

The associations between possible risk factors and the presence of DE were examined using logistic regression. Four variables were tested for association with DE, namely, the age of patients, salivary pH, salivary volume/minute, and salivary pepsin. The following results were obtained: age (odds ratio (OR) = 1.165; 95% confidence interval (CI) = 1.05-1.28), salivary pH (OR = 0.021; 95% CI = 0.001-0.49), salivary volume (OR = 0.225; 95% CI = 0.022-0.73), and salivary pepsin (OR = 1.099; 95% CI = 1.03-1.16), as shown in Table 5.

**Table 5:** Association between dental erosion (total BEWE ≥2) and independent variables using logistic regression analyses. BEWE = Basic Erosive Wear Examination; OR = odds ratio; CI = confidence interval

Independent variables	Dental erosion (Total BEWE ≥2)			
	Wald	P-value	OR	(95% CI for OR)
Age	9.45	0.002	1.16	(1.05–1.28)
Salivary pH	5.74	0.017	0.21	(0.001–0.49)
Salivary pepsin	9.42	0.002	1.099	(1.03–1.16)
Salivary volume/five minutes	6.04	0.014	0.22	(0.022–0.73)

## Discussion

The use of the GerdQ for the diagnosis of GERD in this study was based on Jones et al. [10] who showed that the GerdQ is a useful tool for GERD diagnosis and management at primary healthcare units without the need for a specialist or endoscopy. In this study, GERD was diagnosed endoscopically, and the Los Angeles classification system was used to determine the severity of the condition. This system is comparable with Rath et al. [12] who regard the Los Angeles classification system as the most reliable approach for evaluating esophageal erosions.

In the present study, most GERD patients were in their fourth decade of life (30-39 years), and these findings were similar to those observed in a previous study [13]. Similar to previous studies, we found no association between GERD and age [14]. Although females (55%) in our study suffered from GERD more than males (45%), the association between gender and GERD was not statistically significant. These findings are reinforced by those reported in previous studies [15].

Similar to the present study, these studies found that the relationship between GERD and the consumption of tea or coffee was not statistically significant [16,17]. In this study, we demonstrated no significant difference in GERD prevalence with carbonated beverages. These findings have been reported by previous studies [18]. The analysis showed that GERD was significantly related to the consumption of spicy foods and vinegar. These results have been demonstrated by other studies [19].

The analysis showed that GERD patients were more likely to have DE than healthy subjects. This relationship between GERD and DE has been reported in other studies [20,21].

In this study, the mean value of salivary pH in the GERD group was 6.95 and in the control group was 7.11. In the GERD group, lower salivary pH values were found compared with the control group, and these results agreed with those of other studies [22,23]. On the other hand, some studies disagreed with our findings [24].

The result of this study demonstrated a significantly increased level of pepsin in saliva in patients with GERD (mean = 43.60) compared to the control group (mean = 20.60), which was significant (p < 0.001). Similar observations were reported in other studies [25,26].

In this study, negative associations with DE were seen in relation to salivary pH and salivary volume. These results agreed with a previous study by Ramsay et al. [27]. Positive associations with the presence of DE were seen in relation to salivary pepsin; however, Fisher et al. [6] did not find such an association.

The limitations of this study include the inability to establish a causal association between salivary pepsin and DE due to the limited sample size and the fact that it was only conducted in Ramadi, Iraq.

## Conclusions

The findings of the study suggest that GERD patients had a greater prevalence of DE in addition to significantly higher salivary pepsin levels than healthy subjects. The increase in salivary pepsin levels with gastrointestinal diseases and its role in dental tissue damage in reflux diseases still needs more comprehensive evaluation. Our study suggests that dentists should be aware of the fact that DE in populations exposed to GERD has been increasing considerably.
